# Computational physiological modeling for lung-specific ventilation and perfusion management in *ex vivo* lung perfusion

**DOI:** 10.3389/fphys.2025.1724724

**Published:** 2026-02-03

**Authors:** Daan Imholz, Sue A. Braithwaite, Paul Blankman, Eline Oppersma, Frans H. C. de Jongh, Linda M. de Heer, Bart Luijk, Wolfgang F. F. A. Buhre, Dirk W. Donker, Lex M. van Loon

**Affiliations:** 1 Cardiovascular and Respiratory Physiology, Technical Medical Centre, University of Twente, Enschede, Netherlands; 2 Intensive Care Center, University Medical Center Utrecht, Utrecht, Netherlands; 3 Department of Anesthesiology, University Medical Center Utrecht, Utrecht, Netherlands; 4 Department of Respiratory Medicine, Medisch Spectrum Twente, Enschede, Netherlands; 5 Department of Engineering Fluid Dynamics, University of Twente, Enschede, Netherlands; 6 Department of Cardiothoracic Surgery, University Medical Center Utrecht, Utrecht, Netherlands; 7 Department of Pulmonology, University Medical Center Utrecht, Utrecht, Netherlands

**Keywords:** alveolar dead space, computational physiological model, digital twin, *ex vivo* lung perfusion, intrapulmonary shunt

## Abstract

**Introduction:**

*Ex vivo* lung perfusion (EVLP) relies on standardized ventilation and perfusion protocols to evaluate and preserve donor lungs before transplantation. Yet, these protocols overlook graft-specific physiology, leading to variable dead-space ventilation, intrapulmonary shunting, and increased lung injury.

**Methods:**

We developed and validated a computational physiological model (CPM) of lungs on EVLP. The CPM integrates established principles of lung mechanics, gas exchange, and perfusion with clinical input data. It provides mechanistic insight into *ex vivo* lung physiology and quantifies intrinsic properties such as alveolar dead space and intrapulmonary shunting. Model validation combined in silico experiments to verify physiological coherence with calibration against clinical EVLP data to evaluate predictive performance.

**Results:**

Simulation results closely aligned with clinical measurements of left atrial partial oxygen pressure (root mean squared error (RMSE) of 6.4 mmHg). Sensitivity analysis and uncertainty quantification further elucidated key determinants of oxygen and carbon dioxide dynamics, including the inspired oxygen fraction, intrapulmonary shunt, dead space, and perfusate flow.

**Discussion:**

This CPM enhances understanding of *ex vivo* lung physiology, which may lead to less injurious EVLP management and support safe, extended-duration EVLP.

## 1 Introduction

*Ex vivo* lung perfusion (EVLP) is a technique to perfuse and ventilate donor lungs to assess and extend their suitability for lung transplantation (Van Raemdonck et al., 2023). Suitable donor lungs are scarce, with a wait-list mortality of approximately 30% (Canadian Institute for Health Information, 2024). EVLP offers expansion of the donor pool by testing lungs of marginal quality and facilitating a logistical bridging platform for lungs of standard quality (Ahmad et al., 2022; Braithwaite et al., 2024). Marginal donor lungs are defined by poor oxygenation, abnormalities seen on chest imaging, or pathology found during procurement? When their function stabilizes or improves during EVLP, post-transplant outcomes are comparable to standard quality lungs (Cypel et al., 2011; Divithotawela et al., 2019).

EVLP ventilation and perfusion settings follow standardized protocols, ignoring donor-specific lung properties except for height and ideal weight (Braithwaite et al., 2023). During repeated oxygenation challenges—where FiO_2_ is transiently increased to 100%—these fixed protocols may inadvertently injure certain lungs (Watanabe et al., 2021; Terragni et al., 2016). Experimental studies have shown that during EVLP, alveolar dead space and intrapulmonary shunt volumes can increase to 20% and 30% of total lung volume, respectively (Santini et al., 2021). The absence of a confining chest wall makes non-dependent lung regions prone to hyperventilation and hypoperfusion, while dependent regions experience hypoventilation and hyperperfusion. Both extremes may induce endothelial damage, edema, and inflammation (Protti et al., 2013).

These phenomena reveal that *ex vivo* respiratory physiology remains incompletely understood. Although dozens of ventilation and perfusion parameters are routinely measured, their relationships are difficult to interpret mechanistically and do not explain why some lungs deteriorate on EVLP (Van Raemdonck et al., 2023; Braithwaite et al., 2023). Addressing this requires an integrative physiological framework capable of linking measurements to underlying mechanisms of ventilation–perfusion mismatch and gas exchange.

Therefore, we developed a computational physiological model (CPM) based on established principles of lung mechanics, gas exchange, and perfusion and calibrated it using clinical EVLP data. The CPM quantifies intrinsic properties such as alveolar dead space and intrapulmonary shunting, offering mechanistic insight into *ex vivo* lung physiology. By applying this understanding, EVLP management can be individualized to each donor lung, reducing injury and improving post-transplant function. This work presents the CPM framework, its physiological foundations, and validation through clinical calibration, sensitivity analysis, and uncertainty quantification.

## 2 Methods

### 2.1 Model requirements

**FIGURE 1:**
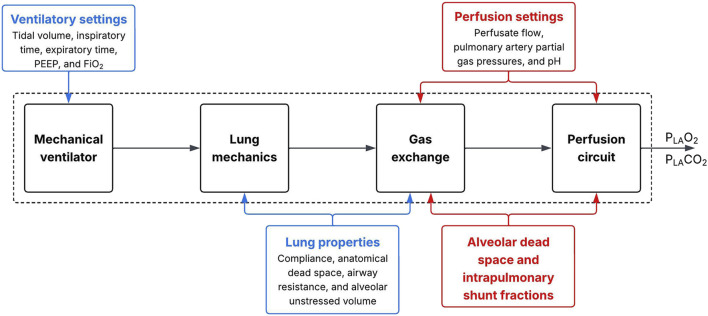
Overview of the four required computational model subcomponents: mechanical ventilator, lung mechanics, gas exchange, and perfusion circuit. The model is indicated by the dotted line with ventilation inputs in blue and perfusion inputs in red. Quantities linking subcomponents are indicated with arrows, with the last arrow indicating the left atrial partial oxygen pressure (P_LA_O_2_) and left atrial partial carbon dioxide pressure (P_LA_CO_2_) as model outputs.

With our CPM of ventilated and perfused lungs in EVLP, we aimed to provide insights into intrinsic lung characteristics, such as alveolar dead space and intrapulmonary shunting. The acellular Toronto protocol formed the basis for model construction (Table 1), and the left atrial partial oxygen pressure (P_LA_O_2_) and left atrial partial carbon dioxide pressure (P_LA_CO_2_) of the perfusate were defined as measurable and interpretable model outputs (Watanabe et al., 2021). The model outputs were derived from the interplay between the three main components during EVLP: a perfusion circuit, a ventilator circuit, and the lung allograft itself. The ventilator circuit was driven by a mechanical ventilator that interacted with the allograft’s lung mechanics, which in turn determined the alveolar gas content available for gas exchange in the alveoli. The interaction between the allograft’s gas exchange functionality and the perfusion circuit determined the left atrial partial gas pressures (Figure 1).

**TABLE 1:** General EVLP protocols for ventilation and perfusion, adapted from Braithwaite et al. (2023).

Setting	Lund	Toronto	Organ care system
Ventilation			
Mode	Volume controlled	Volume controlled	Volume controlled
Tidal volume (ml∙kg^−1^)	6–8	7	6
Frequency (bpm)	10–15	7	10
Positive end-expiratory pressure (cmH_2_O)	5	5	5
Fraction of inspired oxygen (%)	50	21	21
Perfusion			
Target flow	100% of cardiac output	40% of cardiac output	2–2.5 L min^−1^
Pulmonary artery pressure (mmHg)	≤20	≤15	≤20
Left atrial pressure (mmHg)	0 (open left atrium)	3–5	0 (open left atrium)
Pump	Roller	Centrifugal	Piston (pulsatile)
Perfusate	Cellular (red-cell concentrates)	Acellular	Cellular (red-cell concentrates)

All components required a foundation from known physiological principles, interpretable parameters, and normal ranges for an EVLP setting (Table 2). These physiological parameters could either be dictated or measured directly and thereby serve as inputs to the model, or the parameters could describe intrinsic behavior such that they could only be estimated. Physiological parameter variations were required to reflect disease conditions. Model simulations were mandated to capture the behavior of individual ventilator cycles over a total simulation length of multiple minutes.

**TABLE 2:** Overview of model parameters, constants, and outputs with their mathematical symbols, meaning, normal ranges, default values, and units. Normal ranges and default values were based on the *in vivo* literature or on retrospective analyses of EVLP data (Supplementary Material, abbreviated to SM). Oxygen and carbon dioxide are abbreviated to O_2_ and CO_2_, respectively.

Symbol	Meaning	Normal range	Default value	Unit	Source
Clinically measured input parameter					
V_t_	Tidal volume	0.2–0.8	0.5	L	SM
T_insp_	Inspiration duration	1–5	3	s	SM
T_exp_	Expiration duration	2–10	6	s	SM
PEEP	Positive end-expiratory pressure	5–12	6	cmH_2_O	SM
FiO_2_	Inspired O_2_ fraction	0.21–1	0.21	-	SM
C_stat_	Static lung compliance	20–150	80	mL∙cmH_2_O^−1^	SM
Q	Perfusate flow	0.5–3	1.5	L∙min^−1^	SM
H	H^+^ concentration	10^–7.5^–10^–7^	10^–7.3^	-	SM
P_PA_O_2_	PA partial O_2_ pressure	40–100	70	mmHg	SM
P_PA_CO_2_	PA partial CO_2_ pressure	20–45	35	mmHg	SM
Intrinsic parameter					
F_ds_	Alveolar dead space fraction	0–0.95	0.25	-	SM
F_sh_	Intrapulmonary shunt fraction	0–0.95	0.25	-	SM
V_D_	Anatomical dead space volume	0.15–0.35	0.25	L	SM
V_A,0_	Unstressed lung volume	1–2.5	1.5	L	SM
R_aw_	Airway resistance	10–25	15	cmH_2_O∙s L^−1^	SM
Constant					
FiCO_2_	Inspired CO_2_ fraction	-	4.0 × 10^–4^	-	Ben-Tal (2006)
P_atm_	Atmospheric pressure	-	760	mmHg	Guyton and Hall (1996)
P_w_	Water vapor pressure	-	47	mmHg	Hlastala and Berger (2001)
$\dot{V}$ O_2_	Metabolic O_2_ consumption	-	1.5 × 10^–4^	L∙s^−1^	Engoren and Evans (2000), Loer et al. (1997)
$\dot{V}$ CO_2_	Metabolic CO_2_ production	-	1.2 × 10^–4^	L∙s^−1^	Engoren and Evans (2000), Loer et al. (1997)
DO_2_	O_2_ diffusion capacity	-	3.5 × 10^–4^	L∙s^−1^∙mmHg^−1^	Guyton and Hall (1996)
DO_2_′	O_2_ diffusion capacity	-	1.56 × 10^–5^	mol∙s^−1^∙mmHg^−1^	Guyton and Hall (1996)
DCO_2_	CO_2_ diffusion capacity	-	7.08 × 10^–4^	L∙s^−1^∙mmHg^−1^	Guyton and Hall (1996)
DCO_2_′	CO_2_ diffusion capacity	-	3.16 × 10^–5^	mol∙s^−1^∙mmHg^−1^	Guyton and Hall (1996)
σO_2_	O_2_ solubility in perfusate	-	1.4 × 10^–6^	mol∙L^−1^∙mmHg^−1^	Keener (2004)
σCO_2_	CO_2_ solubility in perfusate	-	3.3 × 10^–5^	mol∙L^−1^∙mmHg^−1^	Keener (2004)
V_c,tot_	Total capillary volume	-	7.1 × 10^–2^	L	West (1983)
δ	Acceleration rate	-	10^1.9^	-	Ben-Tal (2006)
r_2_	Dehydration reaction rate	-	0.12	s^−1^	Hill et al. (1973)
l_2_	Hydration reaction rate	-	164 × 10^3^	L∙mol^−1^∙s^−1^	Bidani et al. (1978)
Output					
P_LA_O_2_	LA partial O_2_ pressure	75–150	NA	mmHg	SM
P_LA_CO_2_	LA partial CO_2_ pressure	20–45	NA	mmHg	SM

### 2.2 Conceptual model

**FIGURE 2:**
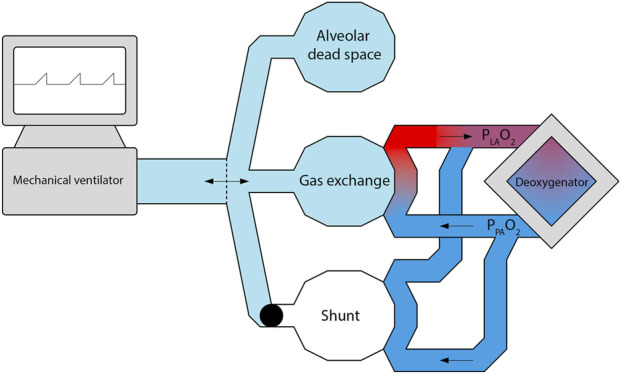
Conceptual model of the EVLP ventilation–perfusion setting. A mechanical ventilator drives airflow into a donor lung represented by three alveolar compartments: gas exchange, alveolar dead space (ventilated but non-perfused), and intrapulmonary shunting (perfused but unventilated). The perfusion circuit, consisting of a centrifugal pump and deoxygenator, delivers perfusate to the pulmonary arteries with a defined oxygen pressure (P_PA_O_2_). After passage through the gas exchange and shunt compartments, the perfusate drains into the left atrium, where the resulting oxygen pressure (P_LA_O_2_) serves as a key model output.

We developed a lumped-parameter CPM to simulate lung ventilation–perfusion inhomogeneity during EVLP. Ventilation–perfusion mismatches were represented by three alveolar volumes: gas exchange volume with matched ventilation and perfusion, alveolar dead space volume with ventilation but no perfusion, and intrapulmonary shunt volume with perfusion but no ventilation. Using a lumped-parameter approach, alveolar volumes were assumed to be spatially homogeneous, with no intratidal recruitment of the shunted volume or blood gas-dependent vascular regulation. The model structure included interconnected components representing the ventilator circuit, *ex vivo* donor lungs, and perfusion circuit (Figure 2). A ventilator generated pressures and gas fractions for the ventilator circuit, while a centrifugal pump drove the flow, and a deoxygenator regulated the partial gas pressures in the perfusion circuit (Watanabe et al., 2021).

### 2.3 Model components

#### 2.3.1 Mechanical ventilator

The ventilator drove the airflow into the respiratory system by generating a positive pressure (P_vent_). Based on the simulated ventilator by Tamburrano et al. (2022), the set tidal volume (V_t_) and positive end-expiratory pressure (PEEP) were linked to the measured static lung compliance (C_stat_) to compute the required plateau pressure (P_plat_) that the ventilator needed to generate (Equation 1).

$$P_{plat}=PEEP+\frac{V_{t}}{C_{stat}}.$$ (1)

In this simplified ventilator, the inspiratory pressure increases linearly from PEEP to the plateau pressure over the set inspiratory time. After inspiration, the ventilator cycles off, and pressure instantly drops to PEEP for the set expiratory time.

#### 2.3.2 Lung mechanics

The *ex vivo* donor lung was split into a mechanical and functional component. The lung mechanics component represented the airflow through the conducting airways and alveolar volume changes. The airways were modeled as a single resistive element (R_aw_), and the alveoli were modeled as an elastic volume defined by the static compliance, as previously described by Chatburn and Faarc (2004) and Albanese et al. (2016). Given the ventilatory pressure as input, the alveolar pressure (P_A_) could be computed as follows Equation 2:

$$\frac{dP_{A}}{dt}=\frac{1}{R_{aw}C_{stat}}\left(P_{vent}-P_{A}\right).$$ (2)

The above simplification of the more complex equation of motion resulted from the absence of a chest wall, pleural pressure, and respiratory muscles in an EVLP setting. The ventilator and alveolar pressure difference drove alveolar volume (V_A_) changes (Equation 3):

$$\frac{dV_{A}}{dt}=\frac{P_{vent}-P_{A}}{R_{aw}}.$$ (3)

These tidal variations in alveolar volume were superimposed on the unstressed lung volume. The alveolar airflow filled the alveolar dead space and gas exchange volumes, with the alveolar dead space volume being linearly proportional to the alveolar dead space fraction (F_ds_). This lung mechanics modeling approach assumed a linear pressure–volume relationship with laminar flow and constant airway resistance and static compliance throughout the ventilatory cycle.

#### 2.3.3 Gas exchange

**FIGURE 3:**
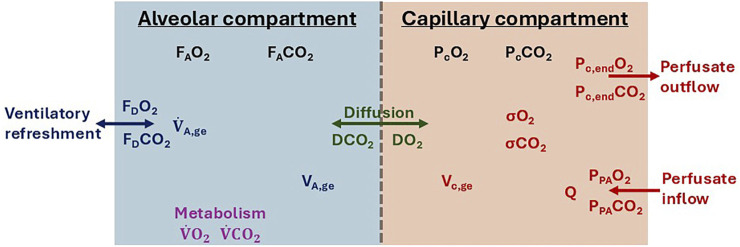
Schematic overview of the components driving the gas exchange between the alveolar and capillary volumes. State variables are indicated in black, ventilatory parameters in blue, lung metabolism parameters in purple, diffusion parameters in green, and perfusion parameters in red. All symbols are described in Table 2.

The functional lung component describes the gas exchange of oxygen and carbon dioxide between the alveolar gas exchange volume (V_A,ge_) and lung capillaries (Figure 3). The alveolar oxygen fraction (F_A_O_2_) and carbon dioxide fraction (F_A_CO_2_) were related to the capillary partial oxygen pressure (P_c_O_2_) and carbon dioxide pressure (P_c_CO_2_), like the conveyor model of Ben-Tal (2006). The alveolar gas fractions were the number of oxygen or carbon dioxide molecules compared to the total number of gas molecules. These fractions changed due to diffusion over the alveolar–capillary membrane, inspiratory refreshment, and lung metabolism (Equations 4, 5):

$$\begin{array}{ll} \frac{dF_{A}O_{2}}{dt} & =\frac{1}{V_{A,ge}}\left(DO_{2}\left(P_{c}O_{2}-P_{A}O_{2}\right)+\left(F_{D}O_{2}-F_{A}O_{2}\right){\dot{V}}_{A,ge,i}\right.\\ & \;-F_{A}O_{2}\left(D{CO}_{2}\left(P_{c}{CO}_{2}-P_{A}{CO}_{2}\right)+DO_{2}\left(P_{c}O_{2}-P_{A}O_{2}\right)\right)\\ & \;\left.+\;\;\left(F_{A}O_{2}-1\right)\dot{V}O_{2}-F_{A}O_{2}\dot{V}{CO}_{2}\right), \end{array}$$ (4)

$$\begin{array}{ll} \frac{dF_{A}{CO}_{2}}{dt} & =\frac{1}{V_{A,ge}}\left(D{CO}_{2}\left(P_{c}{CO}_{2}-P_{A}{CO}_{2}\right)+\left(F_{D}CO_{2}-F_{A}{CO}_{2}\right)\right.\\ & \;{\dot{V}}_{A,ge,i}-F_{A}{CO}_{2}\left(D{CO}_{2}\left(P_{c}{CO}_{2}-P_{A}{CO}_{2}\right)\right.\\ & \;\left.\left.+\;\;DO_{2}\left(P_{c}O_{2}-P_{A}O_{2}\right)\right)+\left(1-F_{A}CO_{2}\right)\dot{V}CO_{2}+F_{A}CO_{2}\dot{V}O_{2}\right), \end{array}$$ (5)

with the diffusion component driven by the alveolar partial gas pressures (P_A_O_2_ and P_A_CO_2_) and diffusion capacities (DO_2_ and DCO_2_) for oxygen and carbon dioxide, respectively (Weibel, 2000). The alveolar partial gas pressures were calculated by multiplying the alveolar gas fractions by the difference between the total alveolar pressure and the water vapor pressure (Table 2; Equations 6, 7):

$$P_{A}O_{2}=F_{A}O_{2}\left(P_{A}-P_{w}\right),$$ (6)

$$P_{A}CO_{2}=F_{A}CO_{2}\left(P_{A}-P_{w}\right).$$ (7)

The inspiratory refreshment depended on the inspiratory alveolar flow to the alveolar gas exchange volume ( $\dot{V}$ _A,ge,i_) and the mixed inspired oxygen and carbon dioxide fractions (F_D_O_2_ and F_D_CO_2_). The mixing effect depended on the size of the anatomical dead space volume and the set inspiratory gas fractions (FiO_2_ and FiCO_2_). Lung metabolism was quantified by the metabolic oxygen consumption ( $\dot{V}$ O_2_) and carbon dioxide production ( $\dot{V}$ CO_2_).

The capillary compartment contained a physiological salt perfusate without red-cell concentrates, limiting absolute oxygen saturation and allowing passive diffusion of oxygen and carbon dioxide over the alveolar–capillary membrane, following Fick’s law for diffusion (Levitzky, 2003). Next to diffusion, the capillary partial gas pressures are influenced by capillary perfusate refreshment, and the capillary partial carbon dioxide pressure also depends on the buffering of carbon dioxide with bicarbonate (Equations 8, 9):

$$\frac{dP_{c}O_{2}}{dt}=\frac{D{O_{2}}^{\prime }}{\sigma O_{2}V_{c,ge}}\left(P_{A}O_{2}-P_{c}O_{2}\right)+\frac{Q}{V_{c,tot}}\left(P_{PA}O_{2}-P_{c,end}O_{2}\right)$$ (8)

$$\begin{array}{ll} \frac{dP_{c}CO_{2}}{dt} & =\frac{D{{CO}_{2}}^{\prime }}{\sigma CO_{2}V_{c,ge}}\left(P_{A}CO_{2}-P_{c}CO_{2}\right)+\frac{Q}{V_{c,tot}}\left(P_{PA}{CO}_{2}-P_{c,end}CO_{2}\right)\\ & \;+\frac{\delta l_{2}hz}{\sigma CO_{2}}-\delta r_{2}P_{c}CO_{2} \end{array}$$ (9)

with the diffusion component depending on the diffusion capacities (DO_2_′ and DCO_2_′), perfusate solubilities (σO_2_ and σCO_2_), and the available capillary volume for gas exchange (V_c,ge_). The continuous perfusate flow (Q) causes refreshment of the capillary volume, meaning that in a fraction of the total capillary volume (V_c,tot_), the end-capillary partial gas pressures (P_c,end_O_2_ and P_c,end_CO_2_) are replaced by the pulmonary artery partial gas pressures (P_PA_O_2_ and P_PA_CO_2_). The buffering of carbon dioxide by bicarbonate depended on the bicarbonate concentration (z), hydrogen concentration (h), buffering acceleration rate (δ), dehydration reaction rate (r_2_), and hydration reaction rate (L_2_) (Equation 10):

$$\frac{dz}{dt}=\delta r_{2}\sigma CO_{2}P_{c}CO_{2}-\delta l_{2}hz.$$ (10)

#### 2.3.4 Perfusion circuit

In the perfusion component, the perfusate flow was split between the shunted and non-shunted capillary compartments, with the shunting linearly proportional to the intrapulmonary shunt fraction (F_sh_) (Hennigs et al., 2022). The perfusate from both compartments was mixed in the left atrium (Equations 11, 12):

$$P_{LA}O_{2}=P_{PA}O_{2}F_{sh}+\left(1-F_{sh}\right)P_{c,end}O_{2},$$ (11)

$$P_{LA}{CO}_{2}=P_{PA}{CO}_{2}F_{sh}+\left(1-F_{sh}\right)P_{c,end}{CO}_{2}.$$ (12)

The pulmonary artery partial gas pressures served as set inputs to the model and were manually adjusted to the desired values by tuning the sweep gas flow through the deoxygenator in the perfusion circuit.

### 2.4 Model parameters

Parameters were divided into input parameters measured clinically during a regular EVLP setup and unknown estimated intrinsic parameters. Default values and normal ranges of model parameters were based on literature and retrospective analysis of EVLP data, and the default values for constants were based on *in vivo* studies (Table 2) (Ben-Tal, 2006; Guyton and Hall, 1996; Hlastala and Berger, 2001; Engoren and Evans, 2000; Loer et al., 1997; Keener, 2004; West, 1983; Hill et al., 1973; Bidani et al., 1978). Because published solubility values for the acellular perfusate are limited, and perfusate electrolyte and total protein compositions are similar to those in plasma, perfusate oxygen and carbon dioxide solubilities were assumed to be equal to those in plasma.

### 2.5 Software implementation

The model was implemented in Python 3.11.9 using the SciPy and NumPy libraries. The set of ordinary differential equations was solved using the Livermore Ordinary Differential Equation (ODE) solver with automatic method switching for stiff and nonstiff problems (Postawa et al., 2020). The tolerance was set to 10^–9^, and the maximal step size to 0.1 as this combination provided numerically stable solutions while maintaining computational efficiency. Visualizations with a sampling grid of 100 Hz were made using the Matplotlib library. Simulations were conducted on a laptop (Intel i5, 8 GB RAM). The computational model code is available from the corresponding author upon reasonable request for academic research use.

### 2.6 Model validation and evaluation

#### 2.6.1 Physiological simulations and clinical calibration

Physiological model behavior and clinical calibration were assessed using static synthetic and dynamic clinical simulations. In each simulation, the initial conditions for the state variables were defined (Table 3), and the simulations were started after the model reached steady state.

**TABLE 3:** Overview of the model’s state variables with their mathematical symbols, meanings, initial values for simulations, and units.

Symbol	Meaning	Initial value	Unit
P_A_	Alveolar total pressure	7	cmH_2_O
F_A_O_2_	Alveolar oxygen fraction	1.8 × 10^–1^	-
F_A_CO_2_	Alveolar carbon dioxide fraction	3.0 × 10^–2^	-
P_c_O_2_	Capillary partial oxygen pressure	125	mmHg
P_c_CO_2_	Capillary partial carbon dioxide pressure	25	mmHg
z	Bicarbonate concentration	1.7 × 10^–2^	mol∙L^−1^

The static simulations for validating the physiological model behavior utilized synthetic data generated from default model parameters (Table 2). Left atrial partial gas pressures were simulated for 30 s. The effect of intrapulmonary shunt and alveolar dead space ratios was visualized using three scenarios. Both ratios were set to 25% of the total lung volume, to 25% and 50% of the total lung volume, and *vice versa*. The effect of perfusate flow was visualized by a fourth scenario in which the perfusate flow was doubled to 3.0 L per minute, with both ratios set to 25% of total lung volume.

The validation of the CPM consisted of simulating a clinical measurement of the left atrial partial oxygen pressure (Table 2). From the retrospectively registered clinical EVLP data of a donor lung on EVLP for a logistical indication, a 15-min interval was selected in which the donor lungs were tested during an oxygenation challenge (FiO_2_ increased to 100%). The instantaneous FiO_2_ increase provided a dynamic perturbation that tested the model’s ability to reproduce transient responses rather than only steady state values. The registered data were sampled every 3 seconds (XVIVO Perfusion System (XPS™); XVIVO, Gothenburg, Sweden), which enabled insertion of clinically measured tidal volume, inspiratory and expiratory times, PEEP, perfusate flow, pH, pulmonary artery partial oxygen pressure, static compliance, and FiO_2_ into the model. Due to a lower sampling frequency of the registered data, the closest data indices to each simulated time point were selected. To visually calibrate the model simulation to the clinical measurements, the airway resistance, anatomical dead space, intrapulmonary shunt fraction, and alveolar dead space fraction were tuned manually. Calibration performance was quantified by the root mean squared error (RMSE) (Equation 13):

$$RMSE=\sqrt{\frac{\sum_{i=1}^{N}\left|\left|P_{LA}O_{2}\left(i\right)-{\hat{P}}_{LA}O_{2}\left(i\right)\right|{|}^{2}\right.}{N}},$$ (13)

with the total number of registered samples (N), the measured left atrial partial oxygen pressure (P^_LA_O_2_(i)), and the simulated left atrial partial oxygen pressure resampled to the measured sampling grid (P_LA_O_2_(i)).

In all simulations, the output measures and tuned parameters were compared with previously described normal ranges (Table 2) to evaluate model plausibility.

#### 2.6.2 Global sensitivity analysis

The global sensitivity analysis using Morris’ elementary effects provided insight into the importance and interactions or nonlinear effects of each parameter (Morris, 1991; Iooss and Lemaître, 2015). Parameters were defined within their normal range, and constants were set within ±50% of their default value (Table 2). Sensitivity analysis was performed for the mean values of left atrial partial oxygen and carbon dioxide pressures during static steady-state simulations and for the response of the clinically simulated left atrial partial oxygen pressure to a FiO_2_ perturbation during an oxygenation challenge. The left atrial partial oxygen pressure showed a mono-exponential response, which was objectified by computing the fitted time constant (τP_LA_O_2_) (Equation 14):

$$P_{LA}O_{2}={\bar{P}}_{LA}O_{2}\left(1-e^{-t/\tau P_{LA}O_{2}}\right),$$ (14)

with the new steady-state level of the left atrial partial oxygen pressure ( $\bar{P}$ _LA_O_2_) and the current time instance (t). The goodness of the mono-exponential fit was evaluated using the coefficient of determination, with 1 indicating a perfect mono-exponential fit and 0 indicating a complete lack of fit (Chicco et al., 2021).

The obtained importances and interactions (nonlinear effects) for all three outputs were individually scaled from 0 to 1. The sensitivity analysis was implemented using Python’s SALib library (Iwanaga et al., 2022). We used 75 trajectories, each representing a randomized path through the parameter space, and a 10-level grid to discretize the parameter space. The results were visualized using a heatmap generated by the Seaborn library.

#### 2.6.3 Uncertainty quantification

For uncertainty quantification, a Monte Carlo approach was employed by resampling the input parameter space based on the probability density functions of the input parameters in each simulation. The probability density functions assumed a normal distribution with the mean set to the parameter’s default value (Table 2) and a standard deviation based on sensor accuracies (Table 4) (Smith, 2014).

**TABLE 4:** Overview of clinically measured input parameters with their mathematical symbols, meanings, standard deviations in measurement based on the reported sensor accuracy, and units.

Symbol	Meaning	Standard deviation	Unit
V_t_	Tidal volume	2.6 × 10^–2^	L
T_insp_	Inspiration duration	5.1 × 10^–2^	s
T_exp_	Expiration duration	5.1 × 10^–2^	s
PEEP	Positive end-expiratory pressure	1.0	cmH_2_O
FiO_2_	Inspired oxygen fraction	1.3 × 10^–2^	-
Q	Perfusate flow	8.5 × 10^–4^	L∙s^−1^
pH	Potential of hydrogen	5.1 × 10^–2^	-
P_PA_O_2_	PA partial oxygen pressure	5.1	mmHg
P_PA_CO_2_	PA partial carbon dioxide pressure	5.1	mmHg

Output uncertainty was quantified by computing statistics on the output distribution of all simulations. Uncertainty quantification was performed for the previously described static steady-state simulations of the left atrial partial oxygen and carbon dioxide pressures using 10,000 simulations, and the dynamic clinically calibrated simulation of the left atrial partial oxygen pressure using 1,000 simulations. The uncertainty in the dynamic response was visualized using confidence bands for the measured FiO_2_ and the measured and simulated left atrial partial oxygen pressures. Uncertainty quantification was implemented using Python’s NumPy and SciPy libraries, and visualizations were generated using the Matplotlib library.

## 3 Results

### 3.1 Physiological simulations and clinical calibration

**FIGURE 4:**
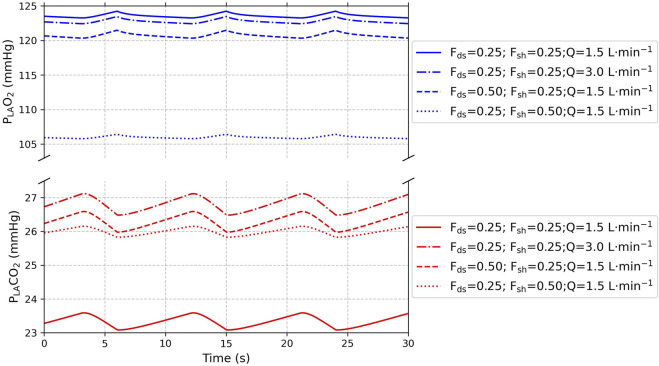
Static simulation of left atrial partial oxygen pressure (P_LA_O_2_) in blue and left atrial partial carbon dioxide pressure (P_LA_CO_2_) in red over a 30-s window with multiple ventilatory cycles. The solid lines represent a 25% alveolar dead space (F_ds_) and 25% intrapulmonary shunt (F_sh_); the dashed lines represent a 50% alveolar dead space and 25% intrapulmonary shunt; and the dotted lines represent a 25% alveolar dead space and 50% intrapulmonary shunt. The dotted–dashed lines represent a doubling of perfusate flow (Q).

The static simulations revealed a respiratory pattern characterized by an increase in left atrial partial oxygen pressure and a decrease in left atrial partial carbon dioxide pressure during the inspiratory phase (Figure 4), and *vice versa* during the expiratory phase, matching physiological ventilation. An increase in alveolar dead space or intrapulmonary shunting resulted in a decrease in the left atrial partial oxygen pressure, with a more profound effect induced by an increase in intrapulmonary shunt fraction. The left atrial partial carbon dioxide pressure increased with an increase in alveolar dead space or intrapulmonary shunting, with a larger effect induced by the alveolar dead space fraction. Doubling the perfusate flow resulted in a decrease in left atrial partial oxygen pressure and an increase in left atrial partial carbon dioxide pressure.

**FIGURE 5:**
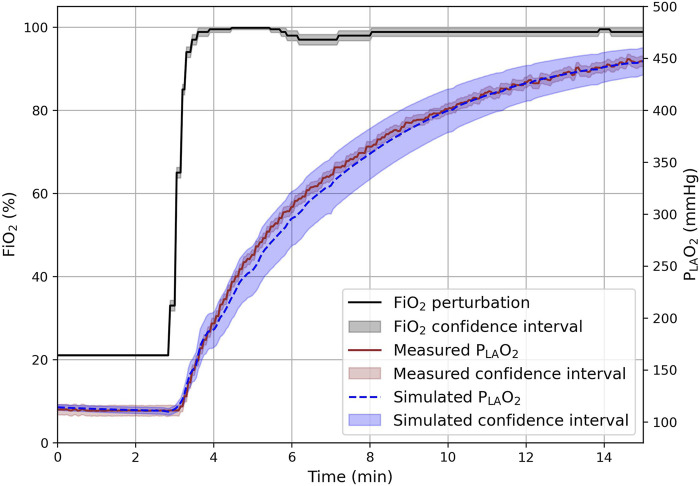
Calibrated clinical simulation consisting of a 15-min epoch with the inspired oxygen fraction (FiO_2_), indicated in black, set to 100% during the oxygenation challenge from 3 min onward. Both the simulated and measured left atrial partial oxygen pressure (P_LA_O_2_), indicated by the dotted blue and solid brown lines, respectively, responded with a delay to the instantaneous increase in the measured inspired oxygen fraction. Confidence intervals were visualized as areas surrounding each variable, each with its own corresponding color.

In the simulated clinical scenario, manual parameter tuning resulted in an airway resistance of 20 cmH_2_O∙s∙L^−1^, an anatomical dead space of 0.29 L, an alveolar dead space fraction of 30%, and an intrapulmonary shunt fraction of 35% of the total lung volume. The simulated left atrial partial oxygen pressure had an RMSE of 6.4 mmHg compared to the clinical measurement (Figure 5). During the 15-min simulation epoch of the oxygenation challenge, the left atrial partial oxygen pressure increased from approximately 100 mmHg to 450 mmHg. The model not only reproduced the new steady state but also captured the delayed, mono-exponential increase in the left atrial partial oxygen pressure following FiO_2_ perturbation, confirming validity under dynamic conditions.

### 3.2 Global sensitivity analysis

**FIGURE 6:**
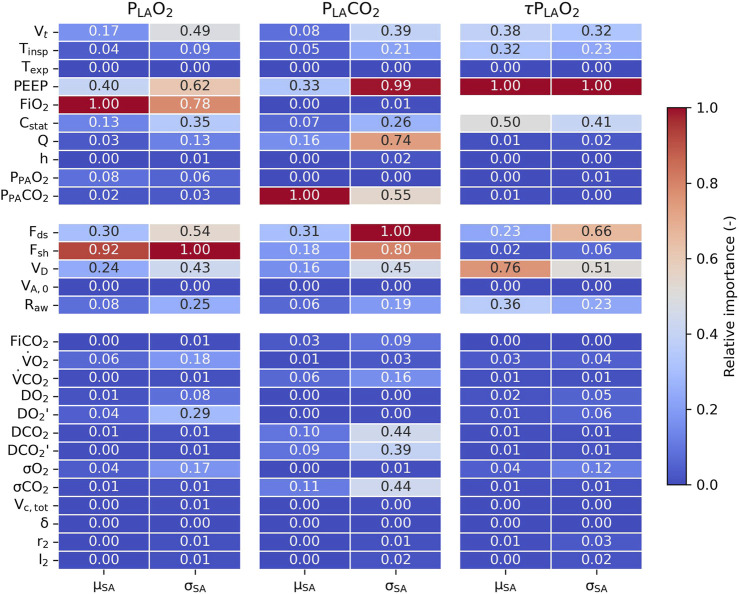
Sensitivity analysis for the steady-state left atrial partial oxygen pressure (P_LA_O_2_), steady-state left atrial partial carbon dioxide pressure (P_LA_CO_2_), and dynamic response of left atrial partial oxygen pressure (τP_LA_O_2_). The relative importance (μ_SA_) and interactions or nonlinear effects (σ_SA_) of all parameters and constants were scaled from 0 to 1, with 1 indicating the most influential parameter.

The global sensitivity analysis indicated FiO_2_ and intrapulmonary shunt fraction as the most determining parameters for the steady-state level of left atrial partial oxygen pressure (Figure 6). In addition, both parameters showed high interactions or nonlinear effects. Other contributing parameters to the left atrial partial oxygen pressure included PEEP, anatomical dead space, and alveolar dead space. The steady-state level of left atrial partial carbon dioxide pressure was predominantly determined by the pulmonary artery partial carbon dioxide pressure, with other contributing parameters including PEEP and alveolar dead space. The largest interactions or nonlinear effects were observed in PEEP, perfusate flow, alveolar dead space, and intrapulmonary shunting. In the dynamic setting, quantified by τP_LA_O_2_ with an almost perfect fit (a median coefficient of determination of 0.99), PEEP and the anatomical dead space were the parameters that contributed the most. Other contributing parameters included airway resistance, static lung compliance, tidal volume, and inspiratory time.

### 3.3 Uncertainty quantification

The model uncertainty yielded a standard deviation of 7.1 mmHg and 3.3 mmHg for the steady states of left atrial partial oxygen and carbon dioxide pressures, respectively (Table 5). The simulated left atrial partial oxygen pressure had a larger uncertainty than the clinically used measurements, whereas the simulated left atrial partial carbon dioxide pressure had a smaller uncertainty (Table 4).

**TABLE 5:** Uncertainty quantification in steady-state simulations for the left atrial partial oxygen pressure (P_LA_O_2_) and left atrial partial carbon dioxide pressure (P_LA_CO_2_) with their mean values, standard deviations, and units.

Simulation	Mean	Standard deviation	Unit
Steady-state P_LA_O_2_	122.5	7.1	mmHg
Steady-state P_LA_CO_2_	24.6	3.3	mmHg

The uncertainty in the clinical simulation of left atrial partial oxygen pressure approximated the measured uncertainty during the initial steady state (4 mmHg vs. 5.1 mmHg; Figure 5). Following FiO_2_ perturbation, simulation uncertainty increased to 22 mmHg as the left atrial partial oxygen pressure increased from ∼100 mmHg to 450 mmHg. Toward the end of the simulation, as the system approached a new steady state, the simulation uncertainty narrowed again to 12 mmHg, while measurement uncertainty remained constant at 5.1 mmHg throughout.

## 4 Discussion

This study presents and validates the first CPM describing ventilation–perfusion dynamics during EVLP. The model integrates clinical EVLP data with fundamental laws of lung mechanics, gas exchange, and perfusion. It reproduced measured left atrial oxygen pressures with high accuracy (RMSE 6.4 mmHg), with all parameters within physiological EVLP ranges. The model exhibited physiologically coherent behavior: oxygenation decreased with higher shunt fractions, carbon dioxide tension increased with larger alveolar dead space, and higher perfusate flow reduced oxygen uptake while augmenting carbon dioxide accumulation (Yeung et al., 2012).

### 4.1 Understanding the physiological framework

FiO_2_ and intrapulmonary shunting had the largest effect on oxygenation as FiO_2_ directly increases alveolar oxygen pressure while intrapulmonary shunting increases the mixing with oxygen-poor perfusate, with the effect of FiO_2_ diminishing with increasing intrapulmonary shunting (Aboab et al., 2006). In contrast, PEEP, alveolar dead space, and anatomical dead space indirectly influence oxygenation. PEEP influences oxygenation by recruiting collapsed alveoli and reducing intrapulmonary shunting, but too much PEEP may also increase alveolar dead space by hyperinflating alveoli and mechanically obstructing perfusate flow. Increases in anatomical or alveolar dead space limit oxygenation by reducing the alveolar volume available for oxygen exchange. Remarkably, the oxygen diffusion capacity, pulmonary artery partial oxygen pressure, and oxygen solubility in the perfusate had a minimal influence on oxygenation. This could be explained by rapid saturation given the perfusate’s low oxygen solubility, resulting in a low absolute oxygen flux across the alveolar–capillary membrane (Yeung et al., 2012). Given the perfusate’s higher carbon dioxide solubility, the perfusate flow, diffusion capacity, and solubility had a greater influence on the left atrial partial carbon dioxide pressure than their oxygenation counterparts. Nonetheless, the balance between carbon dioxide delivery and alveolar clearance was the primary determinant of left atrial partial carbon dioxide pressure (Keogh and Finney, 2008).

The delay in the simulated response to an instantaneous increase in FiO_2_ depends on the interplay between ventilatory settings and lung mechanics, with PEEP, inspiratory time, the tidal volume-to-anatomical dead space ratio, static lung compliance, and airway resistance as determining parameters (Chen et al., 2024). Diffusion over the alveolar–capillary membrane was not a limiting factor, following the fast equilibration of alveolar and dissolved oxygen in the acellular perfusate. Notably, the widening of uncertainty in the simulated left atrial partial oxygen pressure after perturbation reflects the system’s heightened sensitivity to differences in input parameters during a dynamic, nonlinear response. This amplification of input variability into output uncertainty is a well-known phenomenon described in cardiac physiological models (Miram et al., 2016).

### 4.2 Strengths and limitations

Strengths of our CPM include the physiology-based rationale in model construction, yielding a parameter space of physiologically meaningful values within predefined EVLP ranges, with the caveat that in the absence of supporting literature, many normal ranges rely on expert opinion and our non-generalizable retrospective analysis. Another key strength is that validation was performed during dynamic perturbation (FiO_2_ challenge), which is a more stringent test than steady-state comparison alone and confirmed the model’s ability to reproduce transient oxygen dynamics. In addition, EVLP provides a simplified environment devoid of autonomic control mechanisms, allowing us to focus on fundamental ventilation–perfusion dynamics without systemic confounders and making it a uniquely powerful testbed for CPM. Our single-case model evaluation was performed in line with the widely recognized American Society of Mechanical Engineers Verification and Validation 40–2018 Standard for Assessing the Credibility of Computational Models (Assessing the credibility of computational, 2018; Saltelli et al., 2019; Zhang et al., 2020), providing proof-of-concept support for its potential as a clinically applicable physiology-based tool for EVLP. Finally, the model is computationally efficient and suitable for real-time simulation on standard hardware.

Key limitations arise from conceptual assumptions (Fresiello and Donker, 2025), including reliance on *in vivo*-derived constants, restriction to the Toronto protocol, and a zero-dimensional structure that excludes spatial heterogeneity and intratidal (de)recruitment—important mechanisms in ventilator-induced lung injury (Protti et al., 2013). Methodologically, manual parameter tuning to retrospective data introduces a risk of overfitting and non-uniqueness of parameter solutions because multiple parameter sets may yield similar fits within physiological boundaries (Haghebaert et al., 2025).

### 4.3 Clinical implications

The CPM serves as a mechanistic framework for interpreting gas exchange behavior during EVLP. Consequently, the CPM derives functional consequences of donor-specific injury, rather than predictive modeling of biological injury mechanisms. By quantitatively linking clinically measured input parameters to their underlying processes of intrapulmonary shunting, dead space, and perfusate flow, the CPM enables physiological interpretation of observed changes in oxygenation and carbon dioxide dynamics. While conventional parameters, such as lung compliance, airway pressures, pulmonary vascular resistance, and oxygenation, provide a global indication of lung function, they do not distinguish the underlying mechanisms responsible for any impaired gas exchange. The quantification of underlying mechanisms helps differentiate, for example, shunt-driven hypoxemia from dead-space-driven carbon dioxide retention or perfusion maldistribution from inadequate recruitment. The CPM-derived parameters combined with existing clinical metrics may be used to improve the interpretation of oxygenation challenges and refine ventilation and perfusion strategies.

In the case that the CPM derives a large shunt fraction, clinicians may initiate additional investigations to establish the cause of the shunt (such as bronchoscopy to exclude atelectasis or infection and lung ultrasound to observe any edema) and to establish whether the shunt may be improved by interventions such as suction bronchoscopy or proning the lung. In addition, the ventilation strategy may be refined to improve the shunt, including altering PEEP and adjusting the inspiration:expiration time ratio. In the case of alveolar dead space-driven carbon dioxide retention caused by alveolar overdistention, possible refinements may include a decrease in ventilation volumes or, if these are deemed adequate, a slight increase in perfusion flow. Additional diagnostics in the case of extreme levels of dead space may be aimed at excluding pulmonary embolus.

The CPM also provides a physiological basis for evaluating the EVLP protocol (Watanabe et al., 2021). The model further demonstrates that the commonly applied difference between pulmonary artery and left atrial oxygen pressures during oxygenation challenges can be misleading in an acellular perfusate (Ayyat et al., 2020), underscoring the importance of physiology-based assessment criteria. Beyond its immediate explanatory value, the CPM provides a quantitative foundation for future model-driven approaches toward individualized and adaptive EVLP management.

### 4.4 Future development

To enable real-time individualized EVLP management using the CPM’s estimated intrinsic parameters, automatic parameter estimation is required. Given the CPM simulation efficiency and the small number of tunable parameters, an automated, closed-loop calibration procedure with standard optimization algorithms should be developed. Further model refinement should address temperature dependence, spatial resolution to capture regional heterogeneity, and broader ventilation and perfusion modes. These enhancements will increase physiological realism and extend applicability across EVLP protocols. Ultimately, such improvements will form the foundation for future digital twin applications (Venkatesh et al., 2022), enabling reliable parameter estimation from clinical data and personalized EVLP control after broader retrospective and prospective validation. This would result in a digital twin for a ventilated and perfused allograft on EVLP. With the digital twin, the intrapulmonary shunting and alveolar dead space can be estimated, and it also allows for modeling the effects of ventilation and perfusion adjustments during EVLP. The “trial and error” of ventilation and perfusion adaptations would then be evaluated by the digital twin before implementation in the allograft itself.

## 5 Conclusion

We developed and clinically validated the first CPM of ventilated and perfused lungs in EVLP. The model accurately reproduced measured oxygen dynamics and provided mechanistic insights into the roles of intrapulmonary shunting, dead space, and perfusate flow. These insights enhance understanding of the complex EVLP setting and can support refinement of ventilation and perfusion strategies to reduce lung injury. Building on this foundation, the model offers a pathway toward donor lung digital twins for individualized protocols and extended-duration EVLP.

## Data Availability

The original contributions presented in the study are included in the article/Supplementary Material; further inquiries can be directed to the corresponding author.
