# Supplementary material for: Computational physiological modeling for lung-specific ventilation and perfusion management in ex vivo lung perfusion
Source: Front Physiol. 2026 Feb 3;16:1724724. doi: 10.3389/fphys.2025.1724724 (PMC12909158; doi:10.3389/fphys.2025.1724724)
Supplement: Supplementary file 1 [file Supplementaryfile1.docx]

Supplementary Material. Physiological normal ranges in *ex vivo* lung perfusion.

This file contains detailed methods and results supporting the retrospective and literature analysis of normal parameter ranges during *ex vivo* lung perfusion (EVLP).

**Table of contents**

[**1** **Extended methods** 2](#_Toc205898540)

[**2** **Supplementary results** 2](#_Toc205898541)

[**2.1** **Continuous data analysis** 2](#_Toc205898542)

[**2.1.1** **Expiration time** 2](#_Toc205898543)

[**2.1.2** **Inspiration time** 3](#_Toc205898544)

[**2.1.3** **Inspired oxygen fraction** 3](#_Toc205898545)

[**2.1.4** **Left atrial partial oxygen pressure** 4](#_Toc205898546)

[**2.1.5** **Perfusate flow** 4](#_Toc205898547)

[**2.1.6** **pH** 5](#_Toc205898548)

[**2.1.7** **Pulmonary artery partial oxygen pressure** 5](#_Toc205898549)

[**2.1.8** **Positive end-expiratory pressure** 6](#_Toc205898550)

[**2.1.9** **Static lung compliance** 6](#_Toc205898551)

[**2.1.10** **Tidal volume** 7](#_Toc205898552)

[**2.2** **Point-of-care data analysis** 7](#_Toc205898553)

[**2.2.1** **Left atrial partial carbon dioxide pressure** 7](#_Toc205898554)

[**2.2.2** **Pulmonary artery partial carbon dioxide pressure** 8](#_Toc205898555)

[**2.3** **Literature estimations** 8](#_Toc205898556)

[**2.3.1** **Airway resistance** 8](#_Toc205898557)

[**2.3.2** **Alveolar dead space and intrapulmonary shunt fractions** 8](#_Toc205898558)

[**2.3.3** **Anatomical dead space volume** 8](#_Toc205898559)

[**2.3.4** **Unstressed lung volume** 8](#_Toc205898560)

[**3** **Supplementary references** 9](#_Toc205898561)

# **1 Extended methods**

A retrospective analysis was performed on data from EVLP runs conducted at the University Medical Centre Utrecht, the Netherlands, between August 2021 and November 2024. In all EVLP runs, the Toronto protocol was initiated, and in the case of satisfactory results, followed by a period of hypothermic oxygenated perfusion [1]. Only the window during the Toronto protocol was used in our analysis.

Of every EVLP run, the following continuously clinically measured parameters were extracted (XVIVO Perfusion System; XVIVO, Gothenburg, Sweden): expiration time, inspiration time, inspired oxygen fraction, left atrial partial oxygen pressure, perfusate flow, pH, pulmonary artery partial oxygen pressure, positive end-expiratory pressure, static lung compliance, and tidal volume. Point-of-care (POC) samples of the left atrial partial carbon dioxide pressure and pulmonary artery partial carbon dioxide pressure were extracted (i-STAT Alinity; Abbott Point of Care Diagnostics, Princeton, New Jersey, U.S.A.). Continuous parameters were sampled every three seconds, while POC samples were taken approximately every hour.

The analysis for normal ranges was based on the kind of data available. Continuous data were analyzed by treating each EVLP as a separate measured normal distribution into a boxplot. Boxplots from all runs were displayed side by side to show parameter distributions across runs, with their defined normal ranges and default values indicated. POC data were visualized in a single boxplot considering all data over all runs. Lastly, normal ranges of intrinsic parameters were estimated using available literature. All analyses were performed in Python 3.11.9.

# **2 Supplementary results**

The analysis included 28 clinical EVLP runs, of which 17 were indicated for logistical reasons and 11 for marginal indications.

## **2.1 Continuous data analysis**

### **2.1.1 Expiration time**

The normal EVLP range for expiration time yielded 2 to 10 seconds, with a default value of 6 seconds (Supplementary Figure 1). This corresponds to the Toronto protocol’s standard frequency of 7 ventilation cycles per minute with an inspiration:expiration ratio of 1:2.


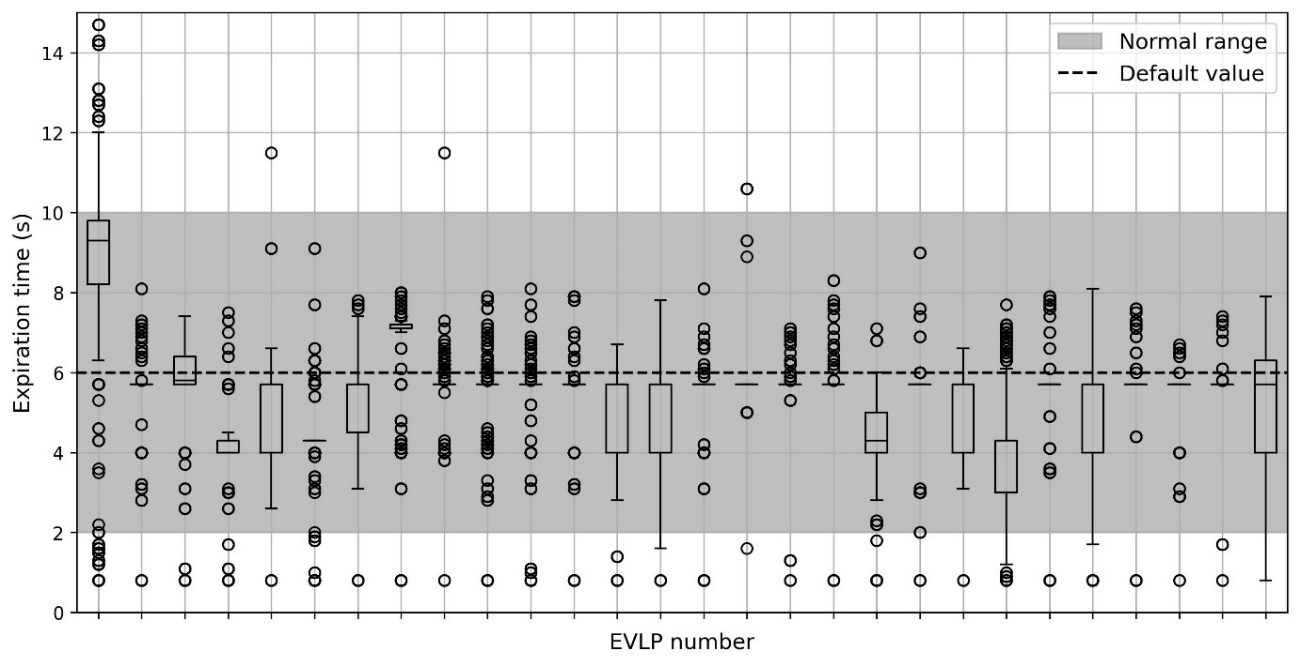


**Supplementary Figure 1.** Distribution of measured expiration time for each EVLP number visualized in a boxplot, with the defined normal range indicated in the grey area and the default value with the dashed black line.

### **2.1.2 Inspiration time**

The normal EVLP range for inspiration time yielded 1 to 5 seconds, with a default value of 3 seconds (Supplementary Figure 2). This corresponds to the Toronto protocol’s standard frequency of 7 ventilation cycles per minute with an inspiration:expiration ratio of 1:2.


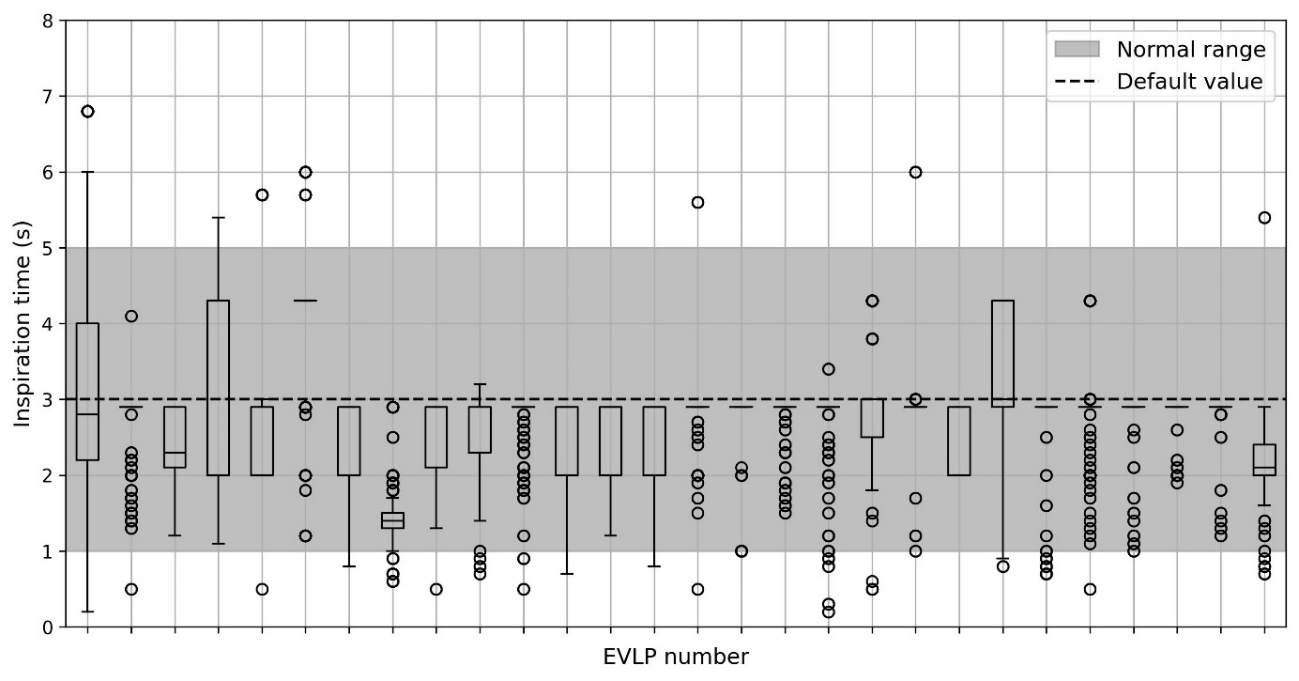


**Supplementary Figure 2.** Distribution of measured inspiration time for each EVLP number visualized in a boxplot, with the defined normal range indicated in the grey area and the default value with the dashed black line.

### **2.1.3 Inspired oxygen fraction**

The normal EVLP range for inspired oxygen fraction yielded 21 to 100 percent, with a default value of 21 percent (Supplementary Figure 3). This follows the Toronto protocol with a standard inspired oxygen fraction of 21 percent, but ranging throughout the entire domain to 100 percent during oxygenation challenges.


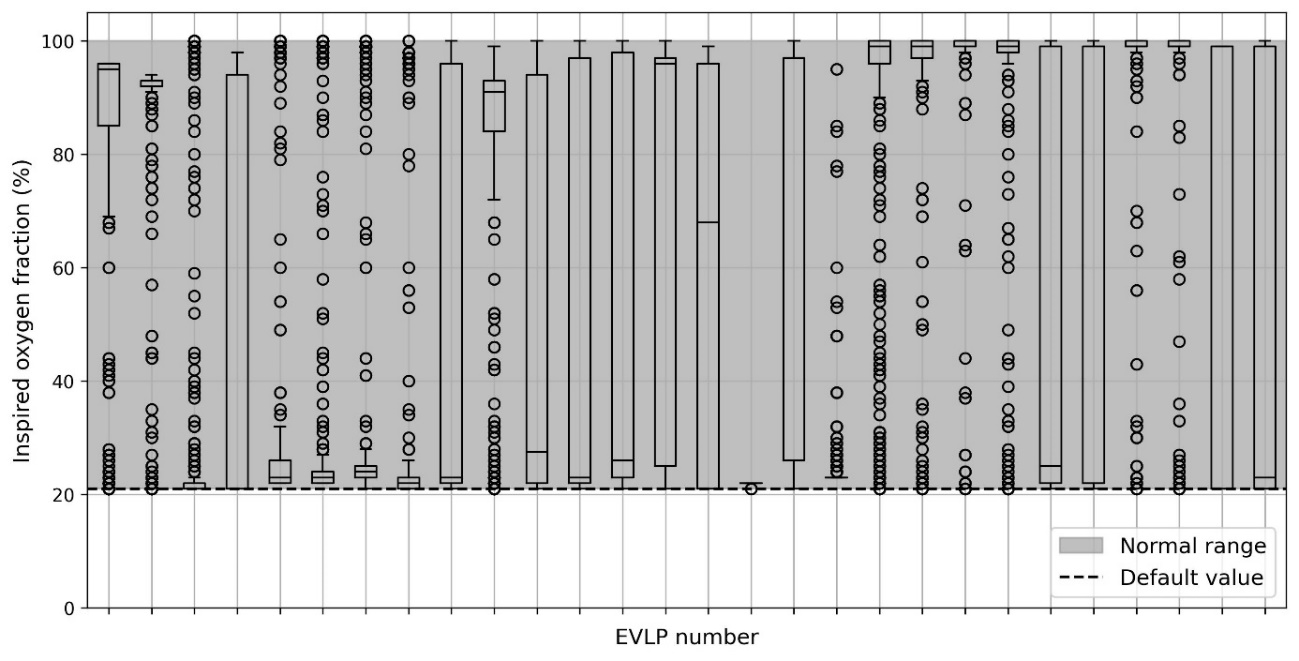
 **Supplementary Figure 3.** Distribution of measured inspired oxygen fraction for each EVLP number visualized in a boxplot, with the defined normal range indicated in the grey area and the default value with the dashed black line.

### **2.1.4 Left atrial partial oxygen pressure**

The normal EVLP range for left atrial partial oxygen pressure yielded 75 to 150 mmHg during normal oxygenation around 21 percent inspired oxygen fraction and normal ventilation. In an oxygenation challenge, the left atrial partial oxygen pressure could increase over a wide domain towards 700 mmHg, visualized by the wide spread in distributions of left atrial partial oxygen pressure (Supplementary Figure 4).


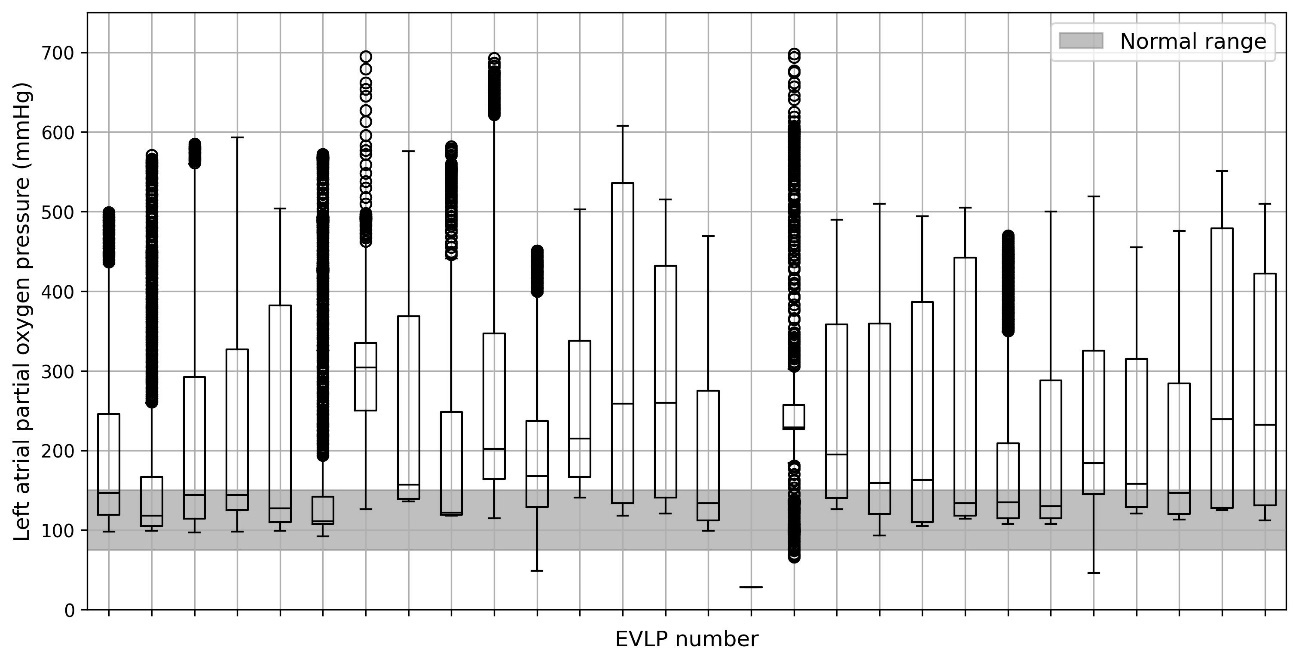


**Supplementary Figure 4.** Distribution of measured left atrial partial oxygen pressure for each EVLP number visualized in a boxplot, with the defined normal range indicated in the grey area.

### **2.1.5 Perfusate flow**

The normal EVLP range for perfusate flow yielded 0.5 to 3 liters per minute, with a default value of 1.5 liters per minute (Supplementary Figure 5). This follows the Toronto protocol, with the standard perfusate flow being 40 percent of normal cardiac output.


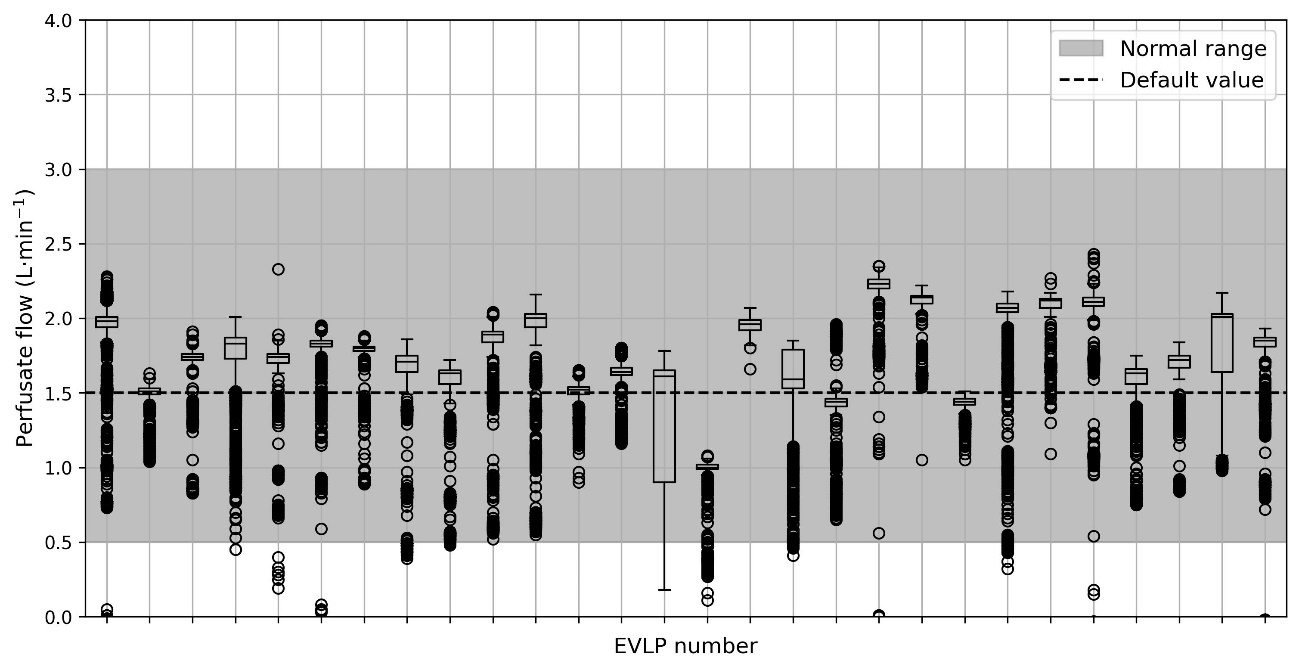


**Supplementary Figure 5.** Distribution of measured perfusate flow for each EVLP number visualized in a boxplot, with the defined normal range indicated in the grey area and the default value with the dashed black line.

### **2.1.6 pH**

The normal EVLP range for pH yielded 7 to 7.5, with a default value of 7.3 (Supplementary Figure 6).


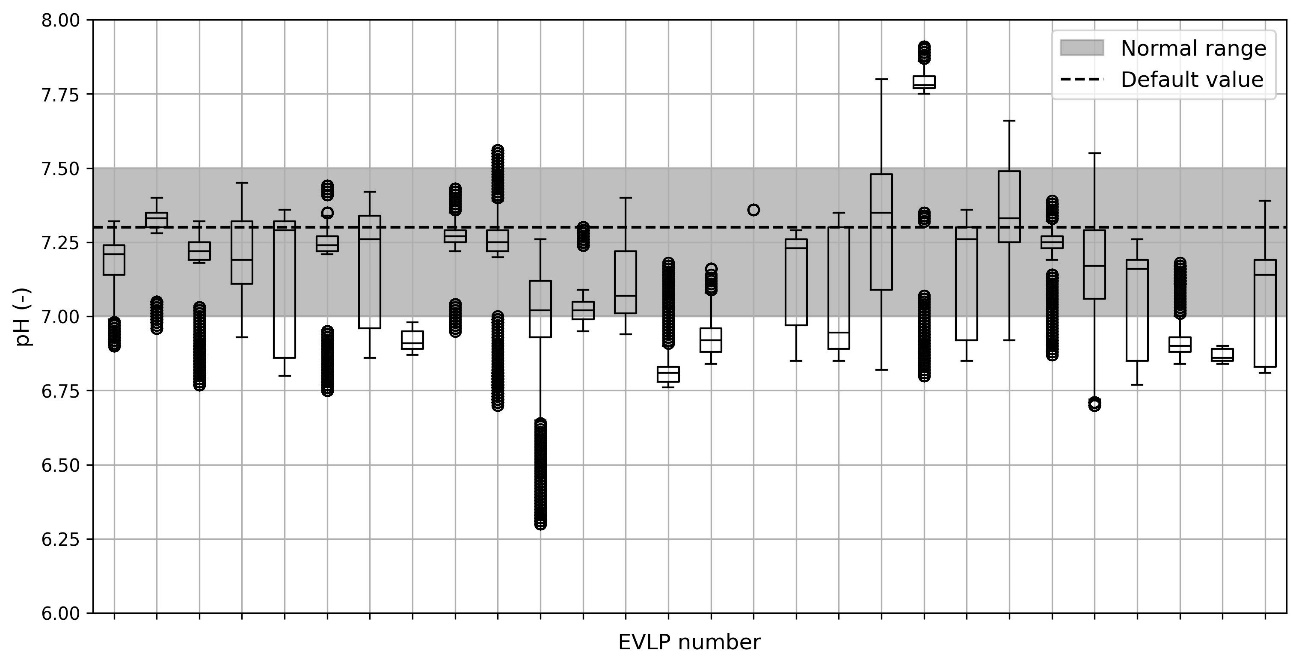


**Supplementary Figure 6.** Distribution of measured pH for each EVLP number visualized in a boxplot, with the defined normal range indicated in the grey area and the default value with the dashed black line.

### **2.1.7 Pulmonary artery partial oxygen pressure**

The normal EVLP range for pulmonary artery partial oxygen pressure yielded 40 to 100 mmHg, with a default value of 70 mmHg (Supplementary Figure 7). The pulmonary artery partial oxygen pressure could be set to each desired value, as it is directly determined by the sweep gas flow. Some distributions include high outliers, indicating a possible measurement error or a delay in changing to sweep gas flow, e.g., during an oxygenation challenge.


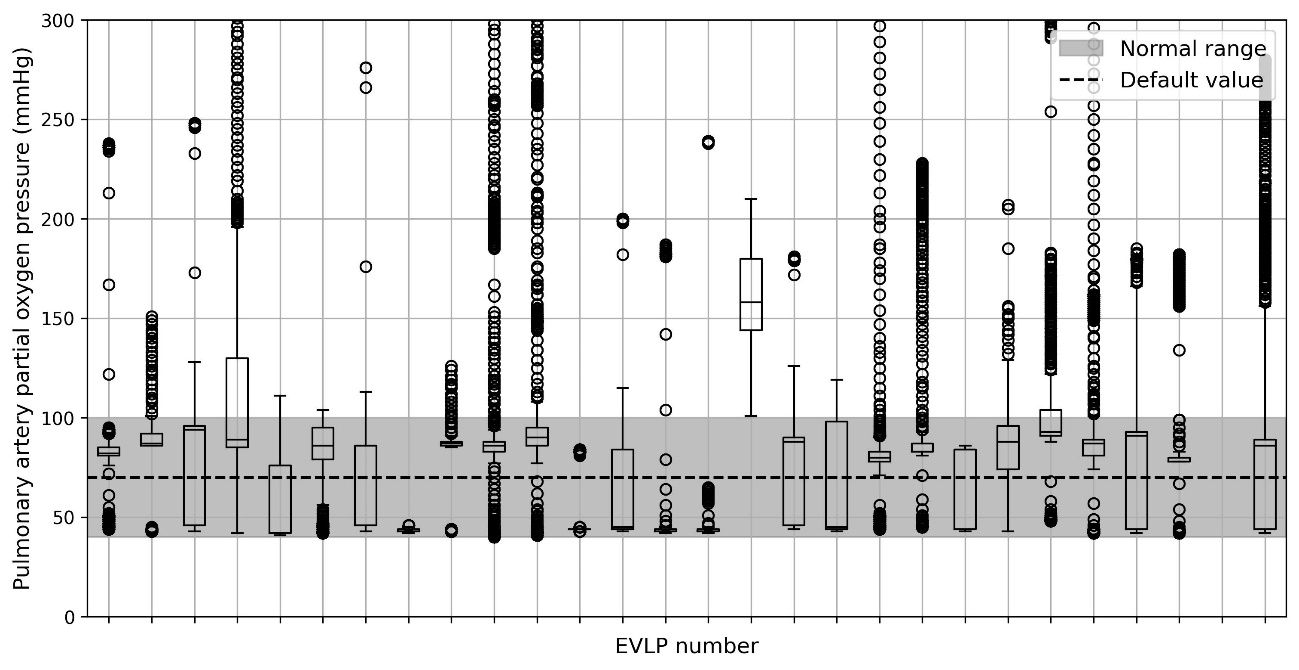


**Supplementary Figure 7.** Distribution of measured pulmonary artery partial oxygen pressure for each EVLP number visualized in a boxplot, with the defined normal range indicated in the grey area and the default value with the dashed black line.

### **2.1.8 Positive end-expiratory pressure**

The normal EVLP range for positive end-expiratory pressure yielded 5 to 12 cmH_2_O, with a default value of 6 cmH_2_O (Supplementary Figure 8). While 5 cmH_2_O is the Toronto protocol’s default positive end-expiratory pressure, a higher positive end-expiratory pressure was used clinically.


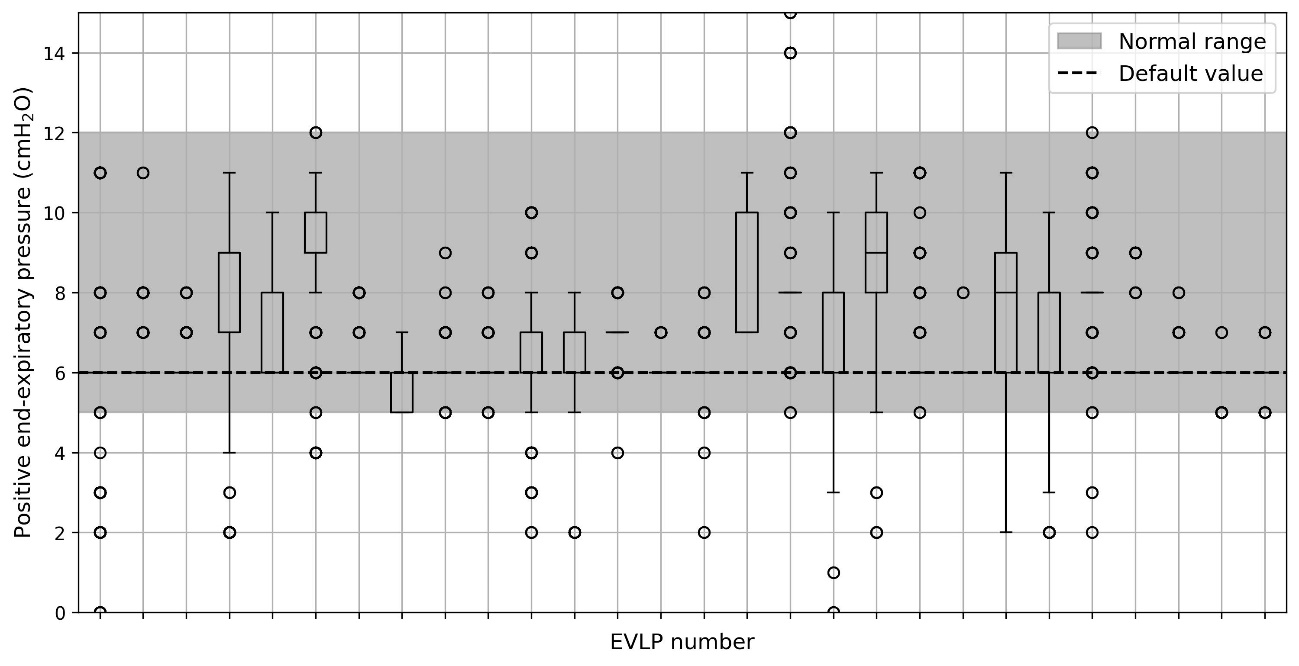


**Supplementary Figure 8.** Distribution of measured positive end-expiratory pressure for each EVLP number visualized in a boxplot, with the defined normal range indicated in the grey area and the default value with the dashed black line.

### **2.1.9 Static lung compliance**

The normal EVLP range for static lung compliance yielded 20 to 150 ml∙cmH_2_O^-1^, with a default value of 80 ml∙cmH_2_O^-1^ (Supplementary Figure 9).


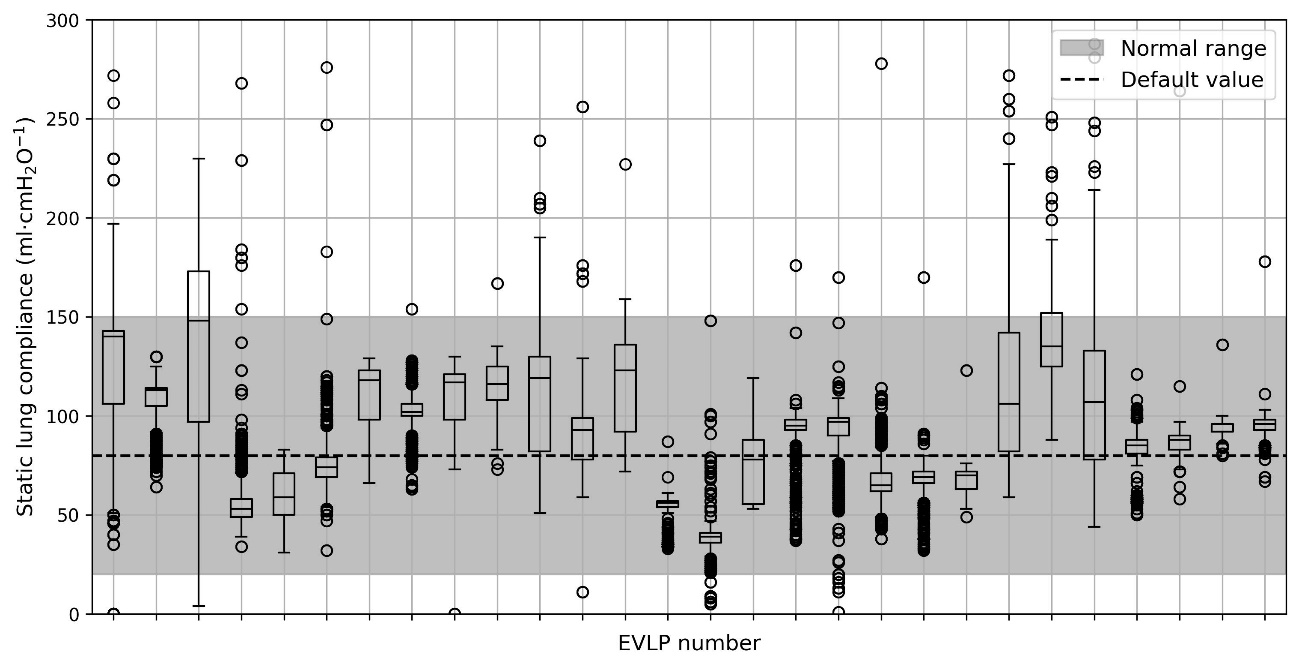


**Supplementary Figure 9.** Distribution of measured static lung compliance for each EVLP number visualized in a boxplot, with the defined normal range indicated in the grey area and the default value with the dashed black line.

### **2.1.10 Tidal volume**

The normal EVLP range for static lung compliance yielded 200 to 800 ml, with a default value of 500 ml (Supplementary Figure 10). This agrees with the Toronto protocol’s default of 500 ml tidal volume.


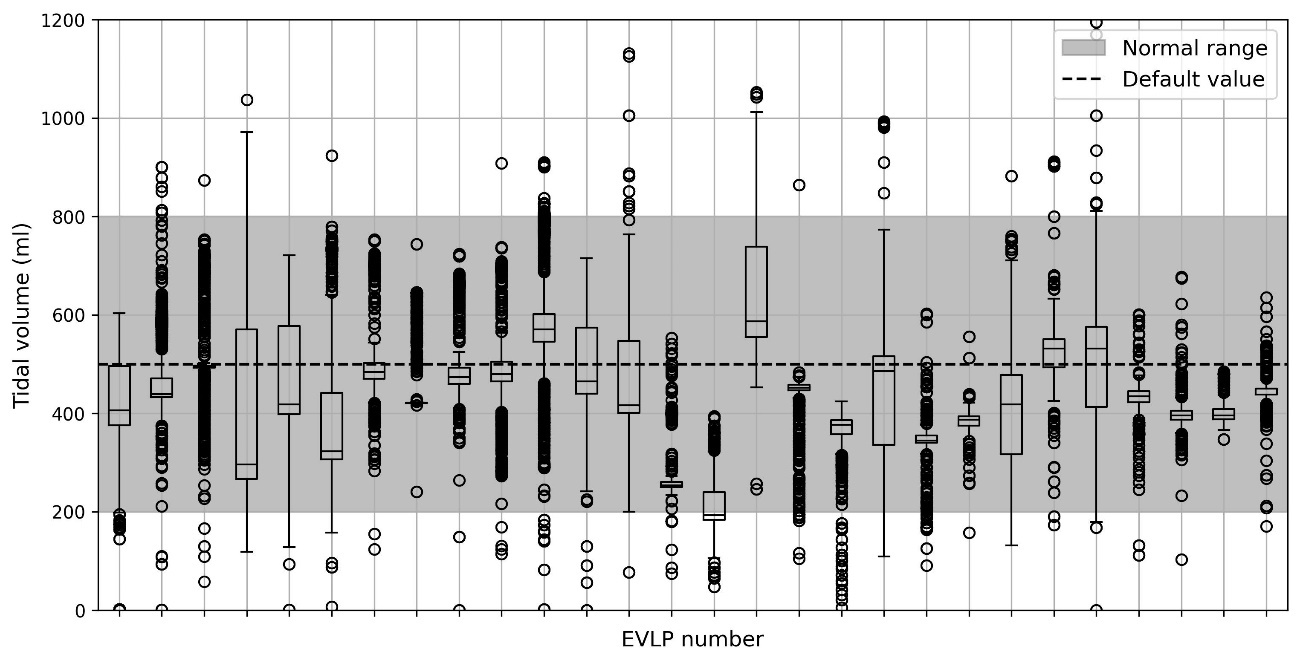


**Supplementary Figure 10.** Distribution of measured tidal volume for each EVLP number visualized in a boxplot, with the defined normal range indicated in the grey area and the default value with the dashed black line.

## **2.2 Point-of-care data analysis**

### **2.2.1 Left atrial partial carbon dioxide pressure**

The normal EVLP range for left atrial partial carbon dioxide pressure yielded 20 to 45 mmHg (Supplementary Figure 11).


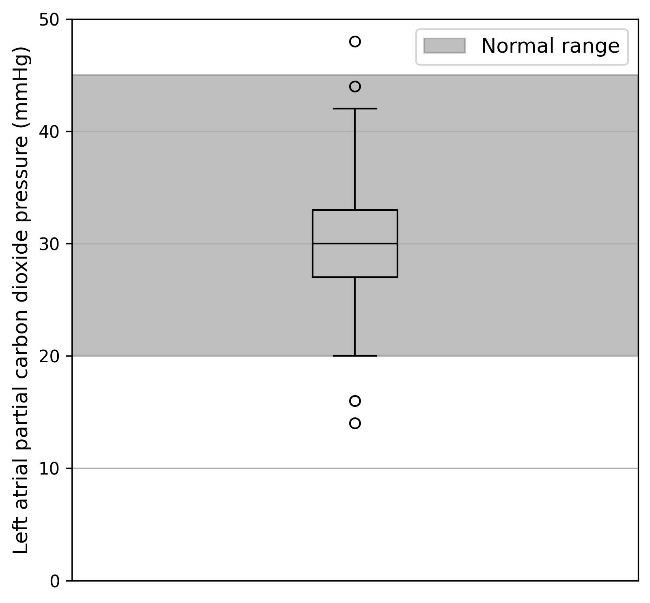


**Supplementary Figure 11.** Distribution of measured left atrial partial carbon dioxide pressure for all point-of-care samples visualized in a boxplot, with the defined normal range indicated in the grey area.

### **2.2.2 Pulmonary artery partial carbon dioxide pressure**

The normal EVLP range for pulmonary artery partial carbon dioxide pressure yielded 20 to 45 mmHg, with a default value of 35 mmHg (Supplementary Figure 12).


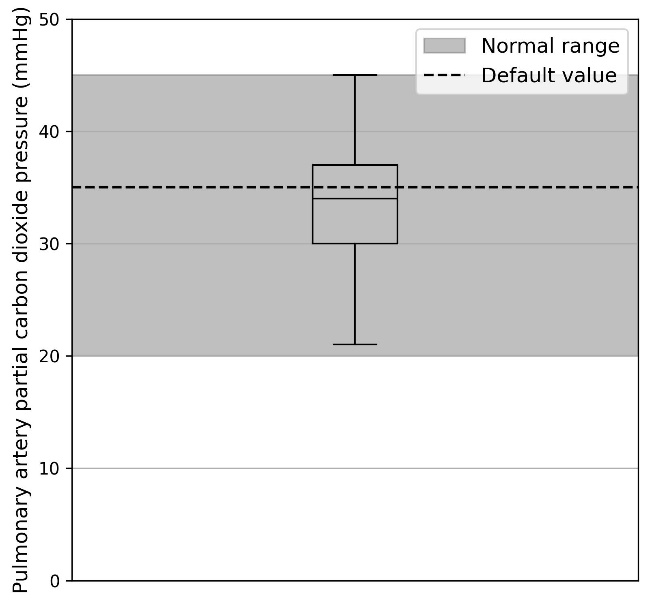


**Supplementary Figure 12.** Distribution of measured pulmonary artery partial carbon dioxide pressure for all point-of-care samples visualized in a boxplot, with the defined normal range indicated in the grey area, and the default value with the dashed black line.

## **2.3 Literature estimations**

### **2.3.1 Airway resistance**

Hamilton Medical describes an airway resistance of 10-15 cmH_2_O∙L^-1^∙s for normal lungs during positive pressure ventilation [2]. Seeing the large apparatus dead space during EVLP, a wide range of 10 to 25 cmH_2_O∙L^-1^∙s, with a default value of 15 cmH_2_O∙L^-1^∙s, was considered.

### **2.3.2 Alveolar dead space and intrapulmonary shunt fractions**

These intrinsic parameters are the unknown metrics that the model provides insight into. Normal ranges are unknown, so the whole range from 0 to 0.95 was considered. As the default value of 0.25 was chosen, given the large fraction of alveolar dead space and intrapulmonary shunt observed during ex vivo ventilation by Santini *et al.* [3].

### **2.3.3 Anatomical dead space volume**

The lower limit of the anatomical dead space was set to an *in vivo* anatomical dead space of 0.15 liters [4]. While the anatomical dead space decreases due to the neglect of the upper respiratory tract in an *ex vivo* setting, the overall anatomical dead space increases due to a large increase in apparatus dead space. In EVLP, a standard 9.0 millimeter endotracheal tube with a tubing length of 1.8 meters is used. Overall, anatomical dead space was considered to be between 0.15 and 0.35 liters, with the default value set at 0.25 liters.

### **2.3.4 Unstressed lung volume**

The unstressed lung volume typically ranges between the residual volume and functional residual capacity. Average values of these volumes range between 1 and 2.5 liters [5], with the default value set to 1.5 liters.

# **3 Supplementary references**

[1] Braithwaite SA, Jennekens J, Berg EM, de Heer LM, Ramjankhan F, de Jong M, et al. Case Report: Optimal utilization of marginal lung allografts by considering donor–recipient PGD risk compatibility and by mitigating allograft and recipient inflammatory risk. Frontiers in Transplantation 2024;3. https://doi.org/10.3389/frtra.2024.1450376.

[2] Arnal J-M. Monitoring respiratory mechanics in mechanically ventilated patients. Hamilton Medical Article 2020.

[3] Santini A, Fumagalli J, Merrino A, Protti I, Paleari MC, Montoli M, et al. Evidence of Air Trapping During Ex Vivo Lung Perfusion: A Swine Experimental Lung Imaging and Mechanics Study. Transplant Proc 2021;53:457–65. https://doi.org/10.1016/j.transproceed.2020.10.016.

[4] Intagliata S, Rizzo A, Gossman W. Physiology, Lung Dead Space. 2025.

[5] Hall GL, Filipow N, Ruppel G, Okitika T, Thompson B, Kirkby J, et al. Official ERS technical standard: Global Lung Function Initiative reference values for static lung volumes in individuals of European ancestry. European Respiratory Journal 2021;57:2000289. https://doi.org/10.1183/13993003.00289-2020.
